# Prevalence and characteristics of chronic musculoskeletal pain in Japan: A second survey of people with or without chronic pain

**DOI:** 10.1007/s00776-013-0525-8

**Published:** 2014-02-07

**Authors:** Masaya Nakamura, Yuji Nishiwaki, Takahiro Ushida, Yoshiaki Toyama

**Affiliations:** 1 Department of Orthopaedic Surgery, School of Medicine, Keio University, 35 Shinanomachi, Shinjuku Tokyo, 160-8582 Japan; 2 Department of Environmental and Occupational Health, School of Medicine, Toho University, Ota, Tokyo, Japan; 3 Multidisciplinary Pain Center, Aichi Medical University, Nagakute, Aichi, Japan

## Abstract

**Background:**

An epidemiological survey conducted in Japan in fiscal year 2010 revealed a high prevalence of chronic musculoskeletal pain, low patient satisfaction with treatment, a high incidence of protracted treatment lasting a year or more, and reduced quality of life. To improve the current system for treating chronic musculoskeletal pain, it is important to identify risk factors, including patient characteristics, for developing chronic pain. Thus, we sought to determine the incidence of new chronic pain in the Japanese population, as well as the persistence rate, associated factors, and current state of treatment of chronic pain, by repeating a postal survey in a nationwide representative sample group first surveyed in 2010.

**Methods:**

Among 11,507 participants in the 2010 epidemiological survey, 1,717 reported chronic pain and 6,283 reported no chronic pain. A repeat questionnaire, mailed to subjects in these 2 groups in fiscal year 2011, received replies from 85 % of those who reported pain and 76 % of those without pain in 2010.

**Results:**

The incidence of new chronic pain was 11.1 %. Risk factors for developing chronic pain included working in a professional, managerial, or clerical/specialist occupation, being female, having a BMI ≥25; currently using alcohol or cigarettes; and having completed an education level of vocational school or higher. Persistent chronic pain was reported by 45.2 % of respondents. Those with severe (VAS score ≥7) and constant lower-back pain lasting more than 5 years had the highest risk of the pain persisting. More than 80 % respondents with persistent chronic pain had a history of treatment, and while about 30 % were still receiving treatment at the time of the survey, the other 50 % had discontinued treatment despite the persistence of pain because of a low degree of satisfaction with treatment.

**Discussion:**

We identified risk factors related to the development of new chronic pain and the persistence of chronic pain. Countermeasures to prevent chronic pain could be especially important for the high-risk populations for understanding the pathology of chronic pain.

## Introduction

The National Livelihood Survey found motor-organ pain in the form of low back pain, stiff shoulders, and arthralgia to be the most common symptoms [1] suffered by the Japanese public. However, we do not know enough about these symptoms, even at a basic level, to create effective strategies to counteract chronic pain in our country. The Survey Study on Chronic Musculoskeletal Pain, conducted in Japan in 2010, found that chronic musculoskeletal pain had a symptom prevalence of 15.4 % and that 42 % of people reporting chronic musculoskeletal pain had received treatment. The treatment period became protracted, lasting a year or more, in 70 % of those who were treated, and patient satisfaction with treatment was low. We also found that chronic musculoskeletal pain strongly impacted the sufferer’s life through both a loss of social activity and a long-term increase in the degree of assistance needed in daily life and also strongly affected the lives of people around the one suffering pain in Japan [2]. This emphasizes the importance of identifying the characteristics and risk factors of patients whose pain becomes chronic, and establishing preventive measures. In the present study, we repeated a postal survey of a representative nationwide sample to examine the incidence of new chronic pain, the chronic pain persistence rate, factors associated with chronic pain, and the actual state of treatment for those with persistent, chronic pain in Japan.

## Methods

**Fig. 1:**
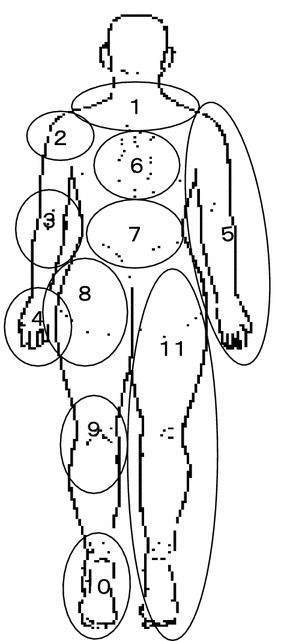
The full-body manikin used in the pain-associated epidemiological survey. *1* neck, *2* shoulder, *3* elbow, *4* wrist/hand, *5* arm, *6* back, *7* low back, *8* hip, *9* knee, *10* ankle/foot, *11* leg

The original survey group, a nationwide, randomly selected sample, was chosen in 2010 through the Mail-in Survey Panel maintained by the Nippon Research Center [2]. The Panel is based on a randomly selected address-based sample with gender and age distributions similar to those in the national population census. To create a mailing address sample that reflected the demographic composition of the Japanese population, subjects were specified as being residents of Japan who were 18 or more years of age, and quotas were set for gender, age, and regional distribution to correspond to the population as a whole. The 2010 survey included 11,507 subjects, of which 1,770 reported chronic pain and the others reported no chronic pain. We mailed a repeat questionnaire to these 2 groups in 2011, and obtained replies from 1,460 of those who had reported chronic pain (reply rate 82.5 %) and 4,797 of those who did not have chronic pain (reply rate 76 %) at the time of the 2010 survey. Besides such basic information as gender, age, location of residence, and occupation, our questionnaire asked about the severity, location, and duration of chronic musculoskeletal pain, whether the pain was treated, and about the facility where treatment was received, the nature of the treatment, the treatment period and effectiveness, and the patient’s degree of satisfaction. In both the 2010 and 2011 surveys, musculoskeletal pain was defined as pain associated with bone, muscle, joints, or nerves at each of 11 anatomical sites (neck, back, low back, shoulder, elbow, wrist/hand, arm, hip, knee, ankle/foot and leg) (Fig. 1), and chronic pain was also defined as pain experienced at least once in the past 30 days, with a severity score of 5 or more on a visual analogue scale (VAS), and persisting for 6 months or more. We calculated the incidence rate of new chronic pain based on the 4,797 persons who did not have chronic pain in fiscal 2010, and the chronic pain persistence rate based on the 1,460 persons who had reported chronic pain in fiscal 2010. Incidence rates and persistence rates were calculated according to the individual factors such as gender, area of residence, and urban size, and occurrence rates were compared by the *χ* ^2^ test. In addition to gender and age, significantly associated factors identified by the crude odds ratio (*p* < 0.1) were ultimately included in multivariate analysis (logistic regression analysis), and adjusted odds ratios were calculated. Factors for which the crude odds ratio did not find an association were also incorporated into the final model, one by one, to check their effect.

We evaluated the treatment circumstances in detail for respondents who reported persistent chronic pain, including whether the pain was treated, the type of treating facility, the nature and effectiveness of the treatment, the subject’s degree of satisfaction, and whether the patient changed treatment facilities. This study was approved by the IRB of Keio University.

## Results

### Incidence rate and risk factors for new chronic pain

Among the 4,797 people who did not have chronic pain in 2010, 531 reported newly developed chronic pain in the 2011 survey; the incidence rate was 11.1 %. Table 1 shows the incidence rates according to individual factors. Crude analysis suggested associations between the development of chronic pain and age, area, city size, occupation, marital status, BMI category, alcohol use, smoking, and education history. Multivariate analysis identified statistically significant associations with gender (female), occupation (professional, managerial, clerical/specialist), a BMI ≥25, current alcohol or cigarette use, and a highest-completed education level of vocational school or higher (Table 1).

**Table 1:** Incidence of chronic pain by factors

		Number	Incidence (%)	Crude OR (95 % CI)	*p* value	Multivariate-adjusted OR^a^ (95 % CI)	*p* value
All		531/4797	11.1				
Gender							
Men		220/2110	10.4	1		1	
Women		311/2687	11.6	1.12 (0.94–1.35)	0.209	1.47 (1.17–1.85)	0.001
Age							
20–29		54/496	10.9	1		1	
30–39		100/733	13.6	1.29 (0.91–1.84)	0.153	1.07 (0.73–1.63)	0.728
40–49		113/794	14.2	1.36 (0.96–1.92)	0.083	1.11 (0.76–1.63)	0.595
50–59		92/794	11.6	1.07 (0.75–1.53)	0.700	0.92 (0.62–1.37)	0.692
60–69		93/1044	8.9	0.80 (0.56–1.14)	0.218	0.80 (0.54–1.20)	0.282
70–79		72/854	8.4	0.75 (0.52–1.09)	0.136	0.89 (0.58–1.35)	0.571
80–		7/82	8.5	0.76 (0.33–1.74)	0.522	0.71 (0.27–1.88)	0.496
Area							
Hokkaido		27/211	12.8	1		1	
Touhoku		32/295	10.9	0.83 (0.48–1.43)	0.501	0.86 (0.50–1.50)	0.602
Kanto		204/1837	11.1	0.85 (0.55–1.31)	0.462	0.80 (0.51–1.23)	0.307
Chubu		55/553	10.0	0.75 (0.46–1.23)	0.256	0.74 (0.45–1.23)	0.246
Hokuriku		17/205	8.3	0.62 (0.32–1.17)	0.138	0.64 (0.33–1.23)	0.182
Kinki		101/855	11.8	0.91 (0.58–1.44)	0.694	0.90 (0.56–1.42)	0.644
Chugoku		38/295	12.9	1.01 (0.59–1.71)	0.977	1.09 (0.63–1.87)	0.760
Shikoku		8/127	6.3	0.46 (0.20–1.04)	0.063	0.52 (0.22–1.19)	0.122
Kyushu		49/419	11.7	0.90 (0.55–1.49)	0.689	0.80 (0.48–1.36)	0.414
City size							
500,000≦		180/1390	13.0	1		1	
150,000≦		163/1521	10.7	0.81 (0.64–1.01)	0.062	0.83 (0.66–1.05)	0.122
<150,000		142/1360	10.4	0.78 (0.62–1.00)	0.041	0.83 (0.65–1.06)	0.134
County		39/401	9.7	0.72 (0.50–1.04)	0.084	0.78 (0.54–1.14)	0.201
No answer		7/125	5.6	0.40 (0.18–1.01)	0.021	0.47 (0.20–1.10)	0.082
Occupation							
Others^b^		346/3427	10.1	1		1	
Professional, manager, clerical, and skill		183/1345	13.6	1.41 (1.16–1.70)	<0.001	1.36 (1.08–1.71)	0.010
Marital status							
Divorced/widowed/single		100/1038	9.6	1		1	
Married		427/3702	11.5	1.22 (0.97–1.54)	0.086	1.27 (0.98–1.64)	0.073
Living condition							
Alone		28/324	8.6	1			
Not alone		497/4417	11.3	1.34 (0.90–2.00)	0.150		
BMI category							
−18.49		48/400	12.0	1.15 (0.83–1.58)	0.395	1.03 (0.74–1.44)	0.864
18.5–24.9		368/3469	10.6	1		1	
25.0–		108/857	12.6	1.22 (0.97–1.53)	0.095	1.28 (1.01–1.62)	0.038
Alcohol drinking^c^							
Never		197/2033	9.7	1		1	
Ex-drinker		49/365	13.4	1.45 (1.03–2.02)	0.031	1.4 (0.98–1.98)	0.061
Current drinker		282/2344	12.0	1.27 (1.05–1.55)	0.014	1.23 (1.00–1.52)	0.050
Smoking^c^							
Never		335/3155	10.6	1		1	
Ex-drinker		74/753	9.8	0.92 (0.70–1.20)	0.524	0.92 (0.69–1.22)	0.567
Current drinker		119/841	14.2	1.39 (1.11–1.74)	0.004	1.32 (1.03–1.69)	0.031
Education							
High school or lower		241/2457	9.8	1		1	
Technical or higher		287/2316	12.4	1.30 (1.08–1.56)	0.005	1.24 (1.02–1.51)	0.030
Income							
−3,990,000		188/1752	10.7	1			
4,000,000–7,990,000		226/2022	11.2	1.05 (0.85–1.29)	0.662		
8,000,000–9,990,000		60/461	13.0	1.24 (0.91–1.70)	0.167		
10,000,000–		48/432	11.1	1.04 (0.74–1.46)	0.820		

^a^ adding to age category and sex, variables which had a statistically significant influence on odds ratio were included in the model

^b^ agriculture, forestry, and fisheries/independent business/part-time worker/full-time homemaker/student/inoccupation

^c^ alcohol drinking and smoking were categorized into three categories [never, ex (used to), and currently smoking] based on the questionnaire

### Persistence rate for chronic pain, and risk factors for persistence

Of the 1,460 persons who reported chronic pain in 2010, 660 reported its persistence in the 2011 survey (45.2 %). Table 2 shows persistence rates according to individual factors. Crude analysis suggested associations between pain persistence and age, area, occupation, marital status, and household income, and the pain site, severity, frequency and duration and change of practice as reported on the 2010 survey. Multivariate analysis identified statistically significant associations with the following factors in the 2010 survey: a pain VAS score of 7–8, constant pain, pain persistence for 5 years or more, and a pain site in the lower back (Table 2). Although the* p* value for the crude analysis of change of practice was 0.082, it is not included in the multivariate analysis because this greatly reduced the sample size. Even if we forcibly included this variable of the model, it did not show a statistically significant result (*p* = 0.299).

**Table 2:** Continuance rate of pain by factors

		Number	Continuance rate	*p* value for *χ* ^2^ test		Crude OR (95 % CI)	*p* value	Multivariate-adjusted OR^a^ (95 % CI)	*p* value
All		660/1460	45.2 %						
Gender									
Men		248/564	44.0 %	*p* = 0.452		1		1	
Women		412/896	46.0 %			1.08 (0.88–1.34)	0.452	1.23 (0.94–1.61)	0.124
Age									
20–29		78/138	56.5 %	*p* < 0.001		1		1	
30–39		125/270	46.3 %			0.66 (0.44–1.00)	0.051	0.74 (0.44–1.24)	0.255
40–49		159/309	51.5 %			0.82 (0.54–1.22)	0.322	1.14 (0.68–1.90)	0.628
50–59		121/269	45.0 %			0.63 (0.42–0.95)	0.028	0.80 (0.47–1.36)	0.411
60–69		101/256	39.5 %			0.5 (0.33–0.76)	0.001	0.76 (0.44–1.33)	0.340
70–79		72/194	37.1 %			0.45 (0.29–0.71)	0.001	0.71 (0.40–1.27)	0.246
80–		4/24	16.7 %			0.15 (0.05–0.47)	0.001	0.37 (0.10–1.30)	0.120
Area									
Hokkaido		32/65	49.2 %	*p* = 0.519		1		1	
Touhoku		41/86	47.7 %			0.94 (0.49–1.79)	0.850	0.96 (0.44–2.07)	0.910
Kanto		264/590	44.8 %			0.84 (0.5–1.39)	0.491	0.64 (0.35–1.18)	0.155
Chubu		85/180	47.2 %			0.92 (0.52–1.63)	0.781	0.81 (0.41–1.60)	0.554
Hokuriku		28/53	52.8 %			1.16 (0.56–2.39)	0.697	0.74 (0.31–1.77)	0.498
Kinki		101/231	43.7 %			0.80 (0.46–1.39)	0.431	0.70 (0.36–1.36)	0.294
Chugoku		33/83	39.8 %			0.68 (0.35–1.31)	0.250	0.55 (0.25–1.21)	0.136
Shikoku		12/39	30.8 %			0.46 (0.2–1.06)	0.067	0.38 (0.14–1.07)	0.067
Kyushu		64/133	48.1 %			0.96 (0.53–1.73)	0.883	0.86 (0.43–1.71)	0.659
City size									
500,000≦		220/460	47.8 %	*p* = 0.605		1			
150,000≦		206/474	43.5 %			0.84 (0.65–1.09)	0.181		
<150,000		173/385	44.9 %			0.89 (0.68–1.17)	0.401		
County		52/114	45.6 %			0.91 (0.61–1.38)	0.672		
Occupation									
Others^b^		491/1139	43.1 %	*p* = 0.002		1		1	
Professional, manager, clerical, and skill		169/319	53.0 %			1.49 (1.16–1.91)	0.002	1.33 (0.96–1.85)	0.086
Marital status									
Divorced/widowed/single		156/287	54.4 %	*p* = 0.001		1		1	
Married		503/1166	43.1 %			0.64 (0.49–0.83)	0.001	0.72 (0.51–1.01)	0.061
Living condition									
Alone		36/70	51.4 %	*p* = 0.292		1			
Not alone		622/1382	45.0 %			0.77 (0.48–1.25)	0.294		
BMI category									
−18.49		63/139	45.3 %	*p* = 0.838		1.02 (0.71–1.46)	0.913		
18.5–24.9		438/977	44.8 %			1			
25.0–		156/334	46.7 %			1.08 (0.84–1.38)	0.552		
Alcohol drinking^c^									
Never		253/591	42.8 %	*p* = 0.240		1			
Ex-drinker		83/169	49.1 %			1.29 (0.92–1.82)	0.146		
Current drinker		322/693	46.5 %			1.16 (0.93–1.45)	0.189		
Smoking^c^									
Never		413/922	44.8 %	*p* = 0.640		1			
Ex-drinker		101/228	44.3 %			0.98 (0.73–1.31)	0.893		
Current drinker		145/304	47.7 %			1.12 (0.87–1.46)	0.378		
Education									
High school or lower		317/715	44.3 %	*p* = 0.540		1			
Technical or higher		339/738	45.9 %			1.07 (0.87–1.31)	0.540		
Income of family									
−3,990,000		220/511	43.1 %	*p* = 0.185		1		1	
4,000,000–7,990,000		280/618	45.3 %			1.1 (0.87–1.39)	0.448	1.00 (0.75–1.34)	0.997
8,000,000–9,990,000		63/149	42.3 %			0.97 (0.67–1.4)	0.867	0.86 (0.55–1.35)	0.510
10,000,000–		80/152	52.6 %			1.47 (1.02–2.11)	0.038	1.14 (0.73–1.78)	0.554
Strength of pain (VAS)									
5–6		412/984	41.9 %	*p* = 0.001		1		1	
7–8		228/433	52.7 %			1.54 (1.23–1.94)	<0.001	1.43 (1.10–1.87)	0.008
9–10		20/43	46.5 %			1.21 (0.65–2.23)	0.547	1.33 (0.63–2.85)	0.455
Frequency of pain									
2–3 times/week		141/404	34.9 %	*p* < 0.001		1		1	
Once/day		100/270	37.0 %			1.1 (0.80–1.51)	0.571	1.34 (0.91–1.96)	0.135
Always		419/786	53.30 %			2.13 (1.66–2.73)	<0.001	2.40 (1.79–3.23)	<0.001
Duration of pain									
<3 years		152/432	35.2 %	*p* < 0.001		1		1	
3–5 years		89/214	41.6 %			1.31 (0.94–1.84)	0.114	1.45 (0.97–2.17)	0.073
5–10 years		145/270	53.7 %			2.14 (1.57–2.91)	<0.001	2.13 (1.47–3.08)	<0.001
10 years–		274/544	50.4 %			1.87 (1.44–2.42)	<0.001	1.76 (1.29–2.42)	<0.001
Site of pain									
Others		81/201	40.3 %	*p* = 0.001		1		1	
Neck		131/252	52.0 %			1.6 (1.1–2.33)	0.013	1.33 (0.87–2.02)	0.188
Shoulder		115/257	44.8 %			1.2 (0.83–1.74)	0.340	1.02 (0.68–1.54)	0.920
Low back		207/393	52.7 %			1.65 (1.17–2.33)	0.004	1.62 (1.11–2.37)	0.012
Knee		32/93	34.4 %			0.78 (0.47–1.3)	0.335	0.81 (0.47–1.39)	0.443
Treatment									
None		342/780	43.9 %	*p* = 0.553		1			
At hospital/clinic		134/289	46.4 %			1.11 (0.84–1.45)	0.462		
At folk remedy		139/295	47.1 %			1.14 (0.87–1.49)	0.336		
Both		26/50	52.0 %			1.39 (0.78–2.46)	0.262		
Change of practice									
No		126/290	43.5 %	*p* = 0.082		1			
Yes		144/284	50.7 %			1.34 (0.96–1.86)	0.082^d^		

^a^ adding to age category and sex, variables which had a statistically significant influence on odds ratio were included in the model

^b^ agriculture, forestry, and fisheries/independent business/part-time worker/full-time homemaker/student/inoccupation

^c^ alcohol drinking and smoking were categorized into three categories (never, ex (used to), and currently smoking) based on the questionnaire

^d^ *p* for crude analysis of change of practice was 0.082, but not included in the multivariate analysis because this reduced sample size

### The state of treatment for persistent chronic pain

#### Characteristics of initial treatment

**Fig. 2:**
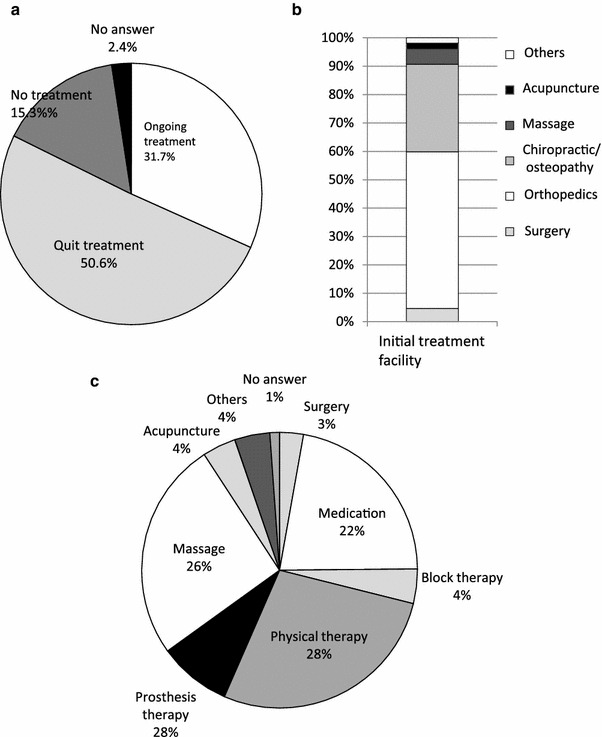
Treatments received for persistent, chronic pain: **a** treatment circumstances, **b** initial treatment facility, and **c** nature of the initial treatment

**Fig. 3:**
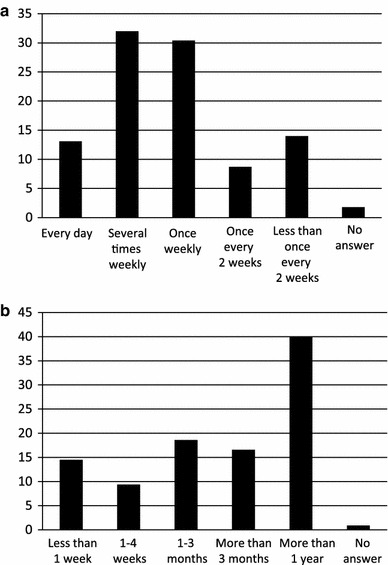
Frequency and duration of treatment for persistent chronic pain: treatment **a** frequency and **b** duration

Although 31.7 % of the people with persistent chronic pain reported ongoing treatment for pain, 50.6 % had received treatment in the past but were no longer being treated, and 15.3 % had never received treatment (Fig. 2a). Approximately 60 % of those with persistent chronic pain and a history of treatment were initially treated at a medical facility such as an orthopaedic surgery department or surgery department, and the others were initially treated with folk medicines such as chiropractic, osteopathy, massage, or acupuncture/moxibustion (Fig. 2b). The most common type of initial treatment was physical therapy (28 %), followed by massage (26 %), medication (22 %), and orthotic treatment (8 %) (Fig. 2c). The most common treatment frequencies were once and several times weekly (approximately 30 % each), followed by once every 2 weeks or less, and daily (Fig. 3a). The most common treatment duration, reported by 40 %, was a year or longer (Fig. 3b).

#### Effectiveness of initial treatment and degree of patient satisfaction

**Fig. 4:**
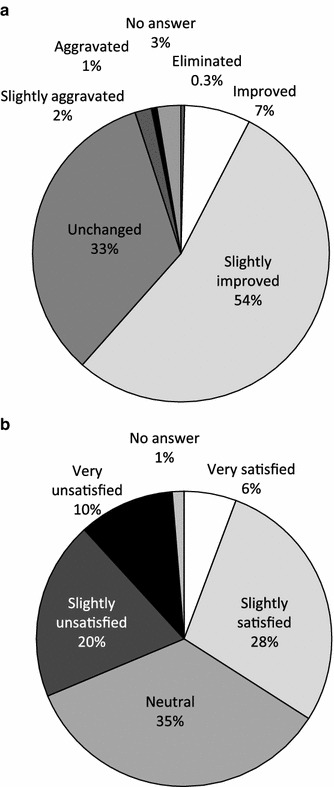
Initial treatment at a medical facility for chronic pain: **a** effectiveness and **b** patients’ degree of satisfaction

**Fig. 5:**
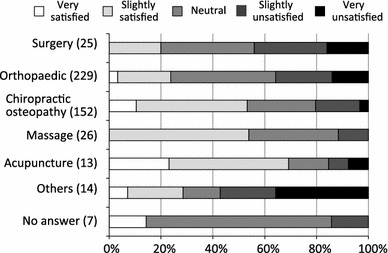
Patient satisfaction with initial treatment, by type of treatment facility

Of the respondents who were initially treated at a medical facility, the pain was improved in 7 %, somewhat improved in 54 %, unchanged in 33 %, somewhat aggravated in 2 %, and aggravated in 1 % by the treatment received (Fig. 4a). Only 6 % reported that they were very satisfied with the treatment received; 28 % were somewhat satisfied, 35 % were neither satisfied nor dissatisfied, 20 % were somewhat dissatisfied, and 10 % were very dissatisfied (Fig. 4b). When compared by the type of treatment provider, 20 % of those treated at medical facilities such as an orthopaedics or surgery department reported being very or somewhat satisfied; however, 50 % of those who used folk medicine such as chiropractic, osteopathy, massage, or acupuncture/moxibustion, reported being very or somewhat satisfied (Fig. 5). Thus, the degree of satisfaction with folk medicine treatments was higher than with treatments received at medical facilities.

#### Circumstances regarding changes in treatment facility

**Fig. 6:**
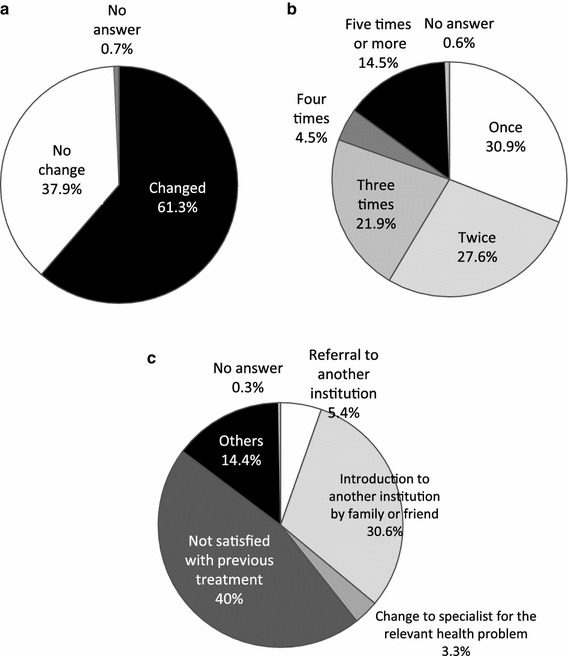
Circumstances of changes in treatment facility: **a** whether changed, **b** number of changes, and **c** reason for changing

Approximately 60 % of the persons who had been treated for pain had changed treatment facilities. Of these, 31 % had changed once, 28 % had changed twice, 22 % had changed 3 times, and, of particular note, a high proportion, 15 %, had changed 5 or more times. The most common reason for changing, given by 40 %, was dissatisfaction with the previous treatment, which is consistent with the low degree of satisfaction reported (Fig. 6).

**Fig. 7:**
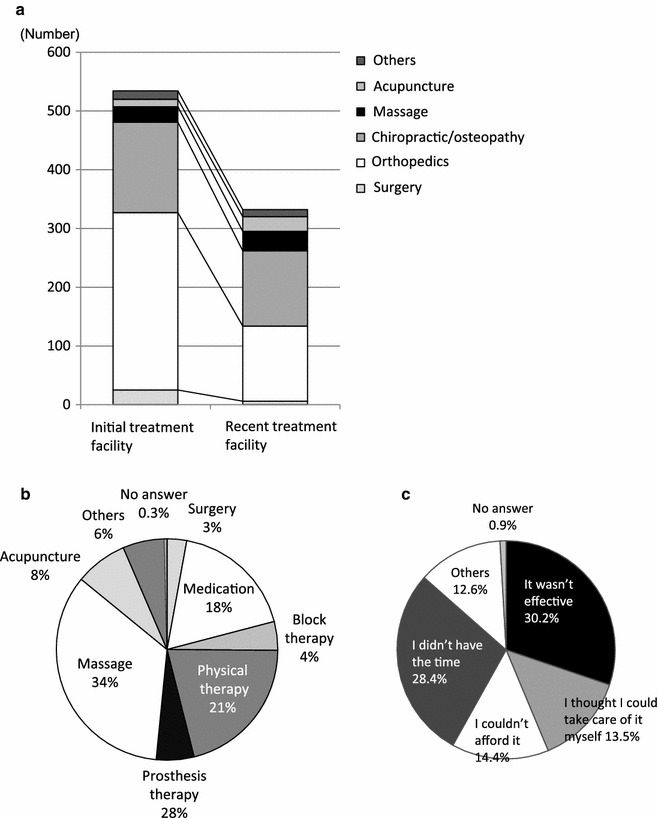
Details of changes in treatment facility: **a** initial and most-recent treatment facility, **b** type of most recent treatment, and **c** reason for discontinuing treatment

A review of the data of the initial and most-recent treatment facilities showed that the use of conventional medical facilities decreased to less than half of the initial frequency, whereas hardly any decrease in folk medicine treatment was observed (Fig. 7a). Reflecting these results, the most common most-recent treatments reported were massage for 34 %, physical therapy for 21 %, and acupuncture/moxibustion for 8 %, thereby accounting for about 60 % of the patients who received treatment. Medication was the most recent treatment for 18 %, nerve block therapy for 4 %, and orthotic treatment for 6 % (Fig. 7b). The most common reason given for discontinuing treatment was, “because it wasn’t effective” (30 %), followed by, “I didn’t have the time,” “I couldn’t afford it,” and, “I thought I could take care of it myself” (Fig. 7c).

#### Actual status of persons with persistent, untreated chronic pain

**Fig. 8:**
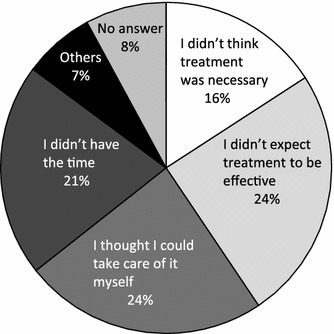
Reasons given for not seeking treatment for persistent chronic pain

Approximately 15 % of the respondents reporting persistent chronic pain had never received treatment (Fig. 2a). The most common reasons given for not seeking treatment were, “I thought I could take care of it myself” (24 %) and, “I didn’t think treatment was necessary” (16 %), indicating inadequate recognition or knowledge of chronic pain. Another 24 % chose, “I didn’t expect treatment to be effective,” indicating a low expectation for successful treatment for chronic pain (Fig. 8). Approximately 40 % of the respondents with untreated chronic pain coped by using non-prescription drugs, health foods, or supplements, or tried to improve their diet or lifestyle.

## Discussion

### New development of chronic musculoskeletal pain

The incidence rate of new chronic musculoskeletal pain among those who did not have chronic pain the previous year was 11.1 %, and in actuality, 1 in 10 persons met the criteria for newly developed chronic pain. On the other hand, the prevalence rate of chronic pain calculated the previous fiscal year was 15.4 %, indicating that much of the chronic pain that met the criteria at that time resolved relatively quickly. Prevalence is generally calculated as prevalence rate = incidence rate × duration of illness; when the corresponding figures were inserted into the equation, the duration of chronic pain was 1.4 years. In other words, according to this calculation, chronic pain resolves in about a year and a half on average. However, this should be interpreted with caution, since it means that the pain no longer meets the criterion for chronic pain after about a year and a half, not that the pain has completely resolved. In addition, caution is required because 48 % of those reporting pain in the 2010 survey said that the pain had persisted for 3 years or longer.

This study identified the following risk factors for the new development of chronic pain: female gender, occupation (professional, managerial, clerical/specialist), a BMI ≥25, current use of alcohol, current use of cigarettes, and completing an education level of vocational school or higher. As many diseases are associated with low socioeconomic status [3], it is very interesting that chronic pain was instead associated with high socioeconomic status, including professional occupations, and higher levels of education. By occupation, managerial, professional, and technical work categories had the highest incidence. The lower back was the most frequently reported site of pain. Previous studies demonstrated that occupational factors, such as long periods of sedentary posture and psychological factors due to dissatisfaction with a work situation, a supervisor, or a dead-end job and boredom, appear to promote the development of new chronic pain [4, 5]. Furthermore, the recent studies demonstrated that the psychosocial factors play important roles in chronic musculoskeletal pain [6–8]. Because the limitation of the present study was that the psychosocial factors were not examined, further study should be performed to clarify the effects of these factors on the chronic musculoskeletal pain in the future. Taken together, consistent with the previous studies [9–12], the relationship between musculoskeletal pain and the identified factors such as female gender, high BMI and smoking may be explained in part by shared risk factors, both physical and psychosocial [13, 14]. The mechanism involved in the current identification of alcohol use as a risk factor for new development of chronic pain is unknown.

### Persistence of chronic musculoskeletal pain

The results showed that 45 % of the respondents who reported chronic pain in 2010 also reported chronic pain in 2011. It is possible that people who suffered from chronic pain through the entire period were more inclined to reply to the second questionnaire; thus, we cannot rule out the possibility that 45 % is an overestimation, even though the reply rate was 85 %. Multivariate analysis did not find any associations between the persistence of chronic pain and basic attributes such as age and gender; the only associated factors were related to the pain itself. A pain severity VAS score of 7–8 was statistically significant. Although the odds ratio increased to 1.30 with the more severe pain reflected in VAS scores of 9–10, it did not reach statistical significance, perhaps because the sample size for this group was so small. The risk of chronic pain persisting a year later was twice as high among persons who had complained of constant pain compared to those who had reported a frequency of 2–3 times a week. The odds ratio for pain persistence was significantly higher for those who reported pain lasting 5 years or more. Based on these findings, those with constant, severe pain persisting 5 years or more appeared to be at the highest risk for the persistence of chronic pain 1 year later. These findings suggested that once the pathological condition of chronic musculoskeletal pain has been established, it could be quite difficult to relieve the chronic musculoskeletal pain. The risk of pain persisting was particularly high for those whose chief complaint was low back pain, compared to pain at other sites. Countermeasures to prevent chronic pain appear to be especially important for these high-risk populations.

### Problems in treating persons with persistent chronic pain and countermeasures

More than 8 out of 10 people with persistent chronic pain had a history of treatment, and while 3 of the 8 were still receiving treatment at the time of the survey, the other 5 had discontinued treatment despite the persistence of pain. Of those who had been treated for pain, 60 % were initially treated at a medical facility; these respondents reported a low degree of satisfaction even though 75 % had received frequent (daily or several times a week) treatment, and 40 % had been treated long-term (a year or more). Of particular note, results by type of treatment provider showed that respondents were less satisfied with treatment received at medical facilities than with folk medicine treatment. We thought that differences in pain severity might be responsible for this finding, but the average VAS scores of those treated at medical facilities and those treated with folk medicine were 6.0 and 5.7, respectively, and this difference was not statistically significant. Other factors might include a tendency toward unrealistically high expectations of medical facilities, and less communication and physical contact in comparison with folk medicine methods. Additional surveys will be necessary in order to verify these factors.

More than 60 % of the respondents with persistent chronic pain had changed their treatment facility; of these, approximately 60 % had changed once or twice. Surprisingly, 15 % of the respondents with persistent chronic pain changed 5 or more times, engaging in so-called “doctor shopping”. A review of the initial and most-recent treatment facilities showed that approximately half of those initially examined in an orthopaedics department changed treatment facilities, but no major change was seen in those initially examined for folk medicine treatment. The results by type of treatment also showed that the use of massage and acupuncture/moxibustion increased, accounting for 42 % of the most-recent treatment types reported. This is consistent with the finding of a low degree of satisfaction with treatment at medical facilities. The recent nationwide survey of chronic pain sufferers in Japan also demonstrated they did not have a high degree of satisfaction with medical treatment [15].

The most common reason given for changing treatment providers or discontinuing treatment was, “because the treatment was ineffective”, which reflects the inadequate effectiveness of the current treatments for chronic musculoskeletal pain. Nociceptive pain, neuropathic pain, and psychogenic pain are intermingled in chronic musculoskeletal system pain, and neuropathic pain is involved in chronic low back pain in particular [16]. Without an adequate grasp of the roles these factors play in the pathology of pain, treatment may fail because it is not appropriate for the patient. Furthermore, the recent studies demonstrated that the psychosocial factors play important roles in chronic musculoskeletal pain [13, 14]. Because the limitation of the present study was that the psychosocial factors were not examined, further study should be performed to clarify the effects of these factors on the chronic musculoskeletal pain in the future.

Many people with persistent chronic pain discontinued treatment. Others did not seek treatment, giving reasons such as not having time, thinking they could take care of it themselves, not thinking they needed treatment, and so on. The majority of the respondents who were not treated for pain reported using non-prescription drugs to cope with the pain. Thus, poor recognition of the seriousness of chronic pain appears to be a problem. It is reported that chronic musculoskeletal pain takes a toll on both mental and physical health, and strongly impacts daily and social life [2]. However, it cannot be said that this state of affairs has been adequately conveyed to the Japanese public. We orthopedists, who specialize in treating the musculoskeletal system, have before us the important task of finding ways to reliably convey the importance of treating chronic pain, to both patients and the general public, through public awareness campaigns.
